# Stroke and Large Vessel Occlusion Risk According to Cincinnati Prehospital Stroke Scale Symptom Patterns in a CPSS-Positive EMS Cohort: A Prospective Cohort Study

**DOI:** 10.3390/jcm15176781

**Published:** 2026-09-01

**Authors:** Filippo Bernasconi, Lorenzo Querci, Michela Generali, Lucia Taurino, Filippo Galbiati, Mariangela Piano, Paola Manzoni, Riccardo Stucchi, Maurizio Migliari, Giuseppe Ristagno, Arturo Chieregato

**Affiliations:** 1 Neuroscience Department, ASST Grande Ospedale Metropolitano Niguarda, 20162 Milan, Lombardy, Italy; lorenzo.querci@ospedaleniguarda.it (L.Q.);; 2 Agenzia Regionale Emergenza Urgenza, 20162 Milan, Lombardy, Italy; m.generali@areu.lombardia.it (M.G.);; 3 Emergency Department, ASST Grande Ospedale Metropolitano Niguarda, 20162 Milan, Lombardy, Italy; 4 Research Department, ASST Grande Ospedale Metropolitano Niguarda, 20162 Milan, Lombardy, Italy

**Keywords:** stroke, large vessel occlusion, emergency medical services, prehospital care, risk assessment, stroke triage, Cincinnati prehospital stroke scale

## Abstract

**Background**: The absolute risk and risk ratio of stroke and large vessel occlusion (LVO) across different Cincinnati Prehospital Stroke Scale (CPSS) categories and symptom patterns remain incompletely defined. This study quantified stroke and LVO risk across CPSS categories, symptom patterns, and predefined CPSS groupings. **Methods**: This single-center prospective observational study included consecutive adults patients assessed by basic life support (BLS) emergency medical service crews in the Milan metropolitan area between March 2023 and December 2024. Patients were eligible if they had a prehospital stroke code based on CPSS ≥ 1, symptom onset within 24 h, a pre-stroke modified Rankin Scale score ≤ 3, and transport to Grande Ospedale Metropolitano Niguarda, a comprehensive stroke center (CSC). Final diagnoses were obtained from hospital records and neuroimaging. Absolute risks and risk ratios for stroke, ischemic stroke, and LVO were calculated across predefined CPSS categories. Discrimination was assessed using receiver operating characteristic analysis. **Results**: Among 876 patients, 411 (47%) had ischemic stroke, 96 (11%) intracerebral hemorrhage, and 164 (19%) LVO. The observed proportion of patients with stroke increased from 41% among those with CPSS = 1 to 78% among those with CPSS = 3, while the corresponding proportion with LVO increased from 6% to 36%. Risk ratios showed a similar gradient. Among patients with CPSS = 2, differences between symptom patterns were small. For LVO, the difference in discrimination between CPSS = 3 and the predefined grouping including CPSS = 3 or CPSS = 2 with Face and Arm deficits was small. Overall, these findings were more consistent with a severity gradient than with superiority of a specific symptom pattern or predefined CPSS grouping. Discrimination across the evaluated CPSS categories and predefined CPSS groupings was limited to modest. **Conclusions**: In CPSS-positive stroke-code patients assessed by BLS crews and transported to a single CSC, higher CPSS scores were associated with progressively higher observed proportions of stroke and LVO. These estimates are conditional on the study cohort and require external validation. They should therefore not be used alone to define transport pathways or guide clinical management.

## 1. Introduction

Early recognition of acute ischemic stroke in prehospital setting remains a major challenge for emergency medical services (EMS) [1,2]. The introduction of endovascular thrombectomy for large vessel occlusion (LVO) has further increased the complexity of prehospital decision making, as clinicians must consider not only the identification of stroke but also the potential presence of LVO when determining initial transport destination [3,4]. This evolution in stroke systems of care has implications for triage protocols, transport times and resource allocation [2,4].

The Cincinnati prehospital stroke scale (CPSS) is the most widely adopted screening tool for prehospital stroke assessment because of its simplicity and validation in basic life support (BLS) settings [5]. Although CPSS demonstrates good sensitivity for stroke detection, its specificity is limited, particularly in everyday BLS practice, resulting in a substantial proportion of false positive activations and stroke mimics [6,7].

Several severity scales have been developed to improve identification of suspected LVO; however, their feasibility and diagnostic performance vary across prehospital systems according to provider training, operational complexity, and local stroke pathways [8,9,10]. Although several prehospital stroke severity scales have been compared in prospective studies, differences in discriminative performance have generally been modest [11]. Moreover, these studies primarily focused on comparisons between different scales rather than on the information conveyed by individual CPSS symptom combinations. Consequently, in many EMS systems, the CPSS remains the primary, and often the only, prehospital stroke assessment tool.

CPSS is traditionally interpreted according to its total score; the diagnostic information conveyed by specific combinations of its individual components has received limited investigation. In particular, the association between specific symptom patterns and the risk of stroke and LVO has been insufficiently explored.

The aim of this study was to quantify the absolute and risk ratio of stroke and LVO across different CPSS symptom patterns and predefined CPSS groupings, and to compare their discriminative ability for prehospital risk stratification in a prospective cohort of patients assessed by BLS EMS crews.

## 2. Methods

### 2.1. Study Design

This was a single-center prospective observational study conducted within the Lombardy regional EMS, involving prehospital missions managed by the Sala Operativa Regionale Emergenza Urgenza (SOREU) Metropolitana. This is one of four emergency medical dispatch centers in Lombardy coordinated by the Agenzia Regionale Emergenza Urgenza (AREU) and has primary responsibility for the Milan metropolitan area. Its operational field covers 450 km^2^, including Milan and its urban belt, and serves just over 2 million residents, corresponding to a population density slightly less than 5000 inhabitants per km^2^. The regional EMS system includes BLS ambulances, Advanced Life Support (ALS) ground units, and Helicopter Emergency Medical Services (HEMS), dispatched according to standardized regional protocols. In general, conscious patients with stable vital signs are initially managed by BLS units, whereas patients with severe impairment of consciousness are directly dispatched to ALS units, irrespective of whether vital signs are abnormal. The study did not modify the prehospital assessment, treatment, or destination decisions, which were performed according to the routine regional EMS pathway.

All BLS personnel in the regional EMS system complete the mandatory AREU training program for emergency medical service providers. This program includes Basic Life Support and Defibrillation (BLSD), trauma care, and instruction in time-dependent emergency protocols, including the recognition of stroke and ST-elevation myocardial infarction. Certification required successful completion of the official AREU examination and was maintained through biennial retraining. The CPSS is a validated prehospital screening tool that assesses three neurological signs: face droop, asymmetric arm drift, and speech disturbance [5]. In this EMS system, CPSS is taught during mandatory provider training and is routinely used in prehospital practice as the standard stroke screening tool. The study was conducted in accordance with the Declaration of Helsinki and approved by the Ethics Committee of Milan Area 2 (14 February 2023; approval No. 85_2023bis). Written informed consent was obtained from each patient prior to enrollment in the study, according to the approved study protocol.

### 2.2. Study Population

All consecutive adult patients (≥18 years old) assisted by BLS crews (i.e., without medical or nursing personnel on board) in the Milan metropolitan area who were assigned a stroke code were transported, according to regional procedure, to the Grande Ospedale Metropolitano Niguarda (GOMN), a Comprehensive Stroke Center (CSC), and were included between March 2023 and December 2024. In the regional EMS pathway, a stroke code is activated for patients who are alert or responsive to verbal stimuli on the AVPU scale, have at least one abnormal CPSS item, have known or presumed symptom onset within 24 h, and have a pre-stroke modified Rankin Scale score ≤ 3. When a stroke code is activated, SOREU Metropolitana prealerts the designated CSC of the incoming suspected stroke patient. Detailed inclusion and exclusion criteria are provided in Supplementary Materials S1. All patients with in-hospital confirmation of the stroke code underwent computed tomography (CT) and CT angiography (CTA) during the same diagnostic assessment.

### 2.3. Sample Size

Study size was determined by the complete consecutive cohort meeting the eligibility criteria, no formal a priori sample-size calculation was performed, as the study was primarily descriptive and was not designed to test a prespecified hypothesis or establish differences in diagnostic performance between CPSS patterns. Confidence intervals were calculated to describe the precision of the reported risk estimates.

### 2.4. Data Collection

Prehospital data were prospectively collected in real time through *EMMA*, the operational management software routinely used by SOREU Metropolitana to record prehospital assessments, clinical findings, treatments, and operational data for each mission. These data were subsequently linked with the emergency department (ED) records from GOMN. For each patient included, the following variables were collected: age, sex, CPSS total score and individual item results, ED discharge diagnosis, and CT and/or CTA reports.

Final diagnoses were obtained from ED records based on clinical assessment and diagnostic imaging performed during the index hospital episode. The records were generated by physicians unaware of the patients’ prehospital CPSS findings, and study investigators subsequently extracted the documented diagnoses and imaging findings. Ischemic stroke was considered a clinical diagnosis and did not require visible ischemic changes on the initial CT. According to the prespecified study protocol, transient ischemic attacks (TIA) and ischemic strokes were recorded within the same diagnostic category. Intracerebral hemorrhage (ICH) was defined by radiological evidence of acute intracranial bleeding on CT. LVO was defined by evidence of an arterial occlusion on CTA, according to the corresponding radiology report, involving the middle cerebral artery (M1 or M2), internal carotid artery, anterior cerebral artery (A1 or A2), posterior cerebral artery (P1 or P2), vertebral artery, or basilar artery. The conventional CPSS score (range 1–3) and individual CPSS symptom patterns were evaluated. Among patients with a CPSS score of 2, symptom patterns were classified as Face + Arm (F + A), Speech + Face (S + F) or Arm + Speech (A + S) according to the combination of neurological deficits. In addition, four predefined CPSS groupings were evaluated: CPSS ≥ 2, CPSS 3 or 2 (F + A), CPSS 3 or 2 (S + F), and CPSS 3 or 2 (A + S).

### 2.5. Statistical Analysis

Stroke and LVO were the primary descriptive outcomes. The primary analysis described outcome risks across the CPSS score categories. Analyses of the individual CPSS = 2 symptom patterns and predefined CPSS-based analytical groupings were supplementary.

Continuous variables are reported as mean ± standard deviation (SD), whereas categorical variables are presented as counts and percentages. Baseline characteristics were summarized overall and according to final outcomes: no stroke, stroke (ischemic stroke + ICH), ICH, ischemic stroke without LVO (non-LVO) and ischemic stroke with LVO.

Absolute risks were calculated as the observed proportion of patients with each outcome within each CPSS score, symptom pattern, or predefined classification rule, and 95% confidence intervals were estimated using the exact binomial method. Risk ratios across the mutually exclusive CPSS total-score categories were calculated using CPSS = 1 as the reference category. For consistency, CPSS = 1 was also retained as a common baseline for the supplementary CPSS-based analytical groupings. For these groupings, the reported estimates quantify risk relative to patients with only one positive CPSS item and do not represent rule-positive versus rule-negative risk ratios. Because the analytical groupings partially overlapped, they were not directly compared with one another. Corresponding 95% confidence intervals were estimated on the log scale using the Wald method and then exponentiated.

Exploratory logistic regression analyses were performed to assess the associations between CPSS configurations and each outcome. Adjusted analyses included gender and age.

Model discrimination was explored using receiver operating characteristic analysis. AUC estimates are reported with 95% confidence intervals. Exploratory comparisons between correlated AUCs were performed using DeLong’s method. Because these analyses were exploratory and no confirmatory conclusions were based on statistical significance, *p* values were not adjusted for multiplicity and should be interpreted descriptively.

Decision-curve analysis was performed as an exploratory analysis to illustrate the hypothetical net benefit of using the evaluated CPSS configurations to identify CPSS-positive patients for an additional LVO-directed action across a range of threshold probabilities [12,13]. It was not intended to reproduce or compare actual transport-destination strategies. Because all participants had already undergone stroke-code transport to a CSC, this analysis cannot establish the clinical utility of these configurations for destination triage and should be considered hypothesis-generating.

Exploratory analyses are reported in the Supplementary Materials (Table S1; Figures S1 and S2).

No imputation of missing data was performed because patients with missing CPSS assessment or unavailable hospital records were excluded before statistical analysis. All analyses were performed using R (version 4.4.1; R Foundation for Statistical Computing, Vienna, Austria). All statistical tests were two-sided, and a *p* value < 0.05 was considered statistically significant. Given the exploratory nature of the inferential analyses, *p* values were interpreted descriptively and not as confirmatory evidence.

## 3. Results

**Figure 1:**
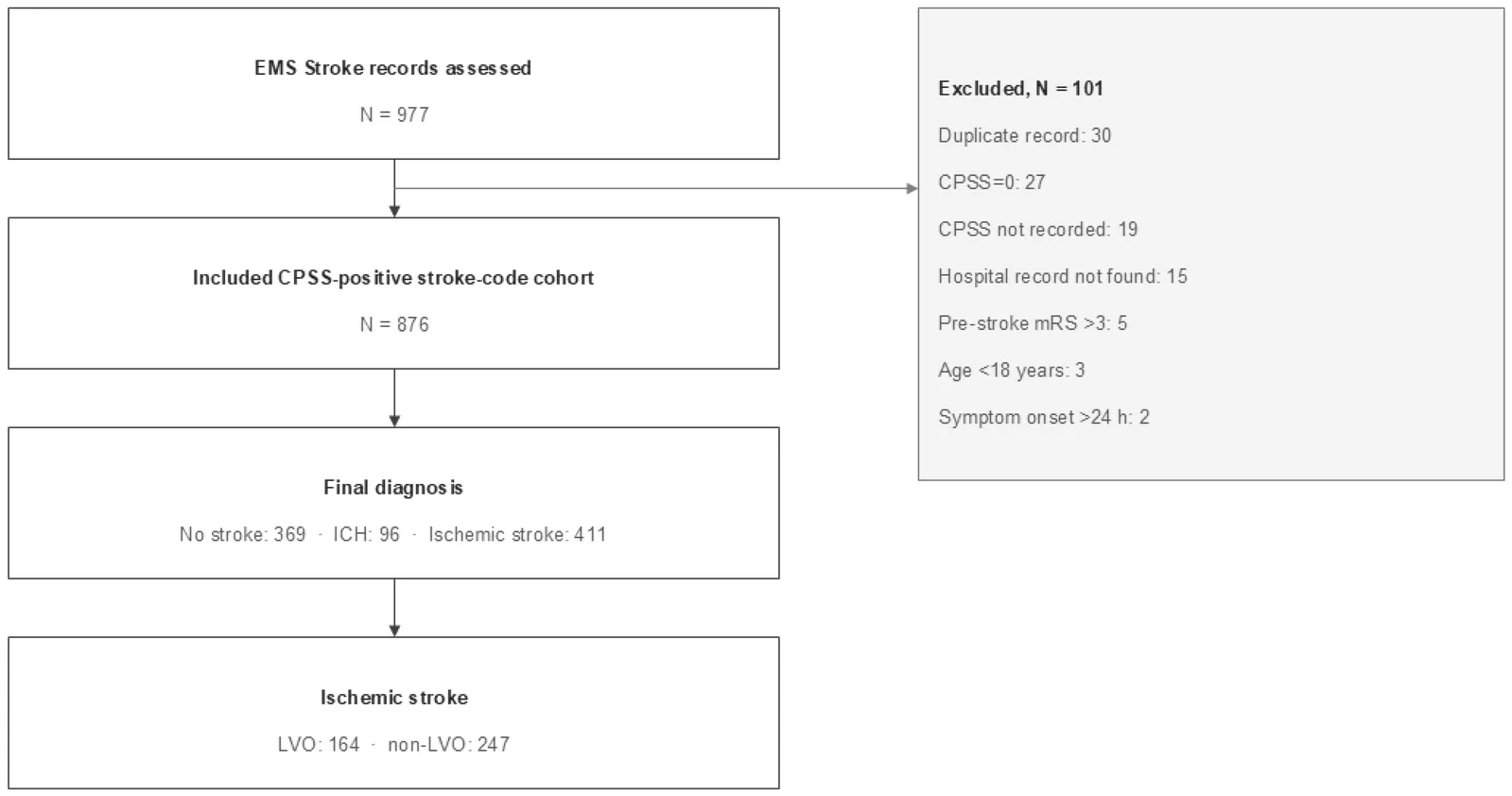
Study population flow. EMS = emergency medical service; CPSS = Cincinnati Prehospital Stroke Scale; ICH = intracerebral hemorrhage; LVO = Large Vessel Occlusion; mRS = modified Rankin scale. The ischemic stroke category includes a small proportion of patients with TIA, as specified in the study protocol.

**Figure 2:**
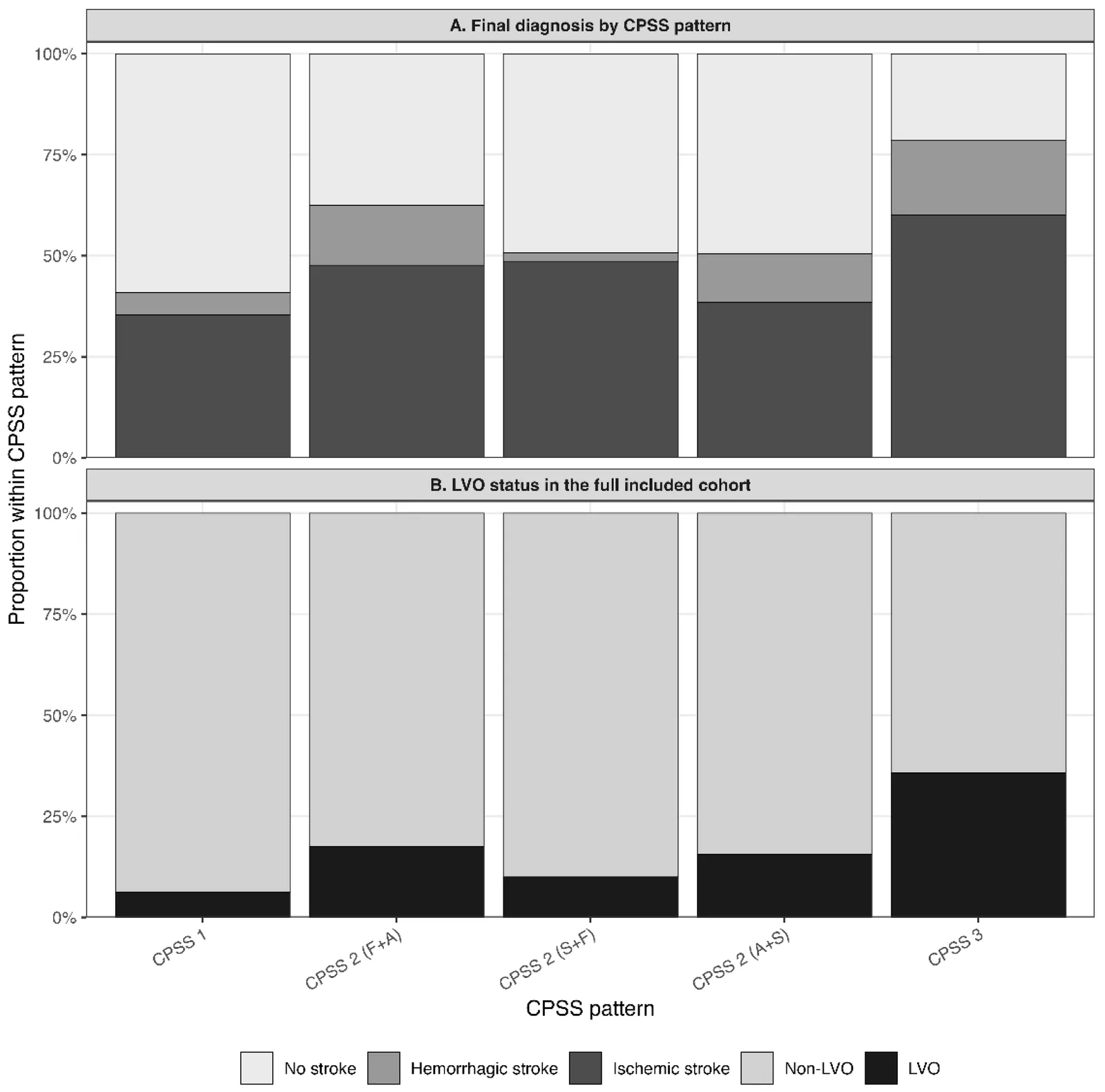
Stacked Bar Charts of CPSS pattern distribution. (**Upper panel**) stroke diagnosis. (**Lower panel**) LVO status. The ischemic stroke category includes a small proportion of patients with TIA, as specified in the study protocol. CPSS = Cincinnati Prehospital Stroke Scale; LVO = Large Vessel Occlusion; F + A = Face droop + Arm drift; S + F = Speech disturbance + Face droop; A + S = Arm drift + Speech disturbance.

A total of 876 patients were included, comprising 369 (42%) no-stroke cases, 96 (11%) ICH, and 411 (47%) ischemic strokes (Figure 1). All patients with ischemic stroke underwent CT and CTA. LVO was identified in 164 patients (19% of the overall cohort), representing 40% of all ischemic strokes (Table 1, Figure 2).

CPSS scores, symptom patterns, and four predefined CPSS groupings showed distinct distributions across diagnostic categories. Patients presenting with a CPSS 1 were more frequently diagnosed with no stroke conditions than with stroke. Among patients with CPSS = 2, the “Speech + Face” symptom pattern was associated with the highest proportion of no stroke diagnoses, whereas the “Face + Arm” pattern showed the highest proportion of stroke. In contrast, CPSS = 3 predominated among patients with ischemic stroke, particularly those with LVO, and among patients with ICH. Similarly, the CPSS 3 or 2 (F + A) and CPSS 3 or 2 (A + S) groupings, both incorporating arm drift, identified a greater proportion of patients with LVO than the CPSS 3 or 2 (S + F) grouping (Figure 2).

Overall, 69% of patients presented with a CPSS score ≥ 2. Absolute and risk ratios across CPSS scores, symptom patterns, and predefined CPSS groupings are reported in Table 2, Table 3 and Table 4. Stroke risk increased from 41% among patients with CPSS = 1 to 78% among those with CPSS = 3, while LVO risk increased from 6% to 36%, respectively. Diagnostic performance of the predefined CPSS groupings across clinical outcomes is summarized in Table 5. For stroke, CPSS ≥ 2 showed the higher sensitivity (78.3%), whereas CPSS = 3 showed the higher specificity (83.2%) and PPV (78.5%), compared with other groups. For LVO, CPSS ≥ 2 showed the higher sensitivity (89.6%), whereas CPSS = 3 showed the higher specificity (74.0%) and PPV (35.8%) than the others. For LVO, the Youden index was 0.361 for the CPSS 3 or 2 (F + A) grouping and 0.368 for CPSS = 3. Detailed results of the logistic regression, ROC, and DCA are provided in the Supplementary Materials. Exploratory logistic regression analyses adjusted for age and sex were consistent with the crude analyses and did not materially change the direction or magnitude of the observed associations (Table S1; Figures S1 and S2).

## 4. Discussion

The principal finding of this study is that CPSS categories identify progressively increasing risks of stroke and LVO, while predefined symptom patterns and predefined CPSS groupings provide additional information for prehospital risk stratification. Patients with CPSS = 1 had a relatively low probability of stroke (41%) and LVO (6%), whereas those with CPSS = 3 had substantially higher risks (78% and 36%, respectively), with risk ratios showing a similar gradient. Together, these findings indicate that CPSS severity conveys information beyond a simple positive-or-negative screening result.

The prevalence of ischemic stroke (47%) and ICH (11%) in our cohort was broadly consistent with that reported in previous prospective EMS studies evaluating patients with suspected stroke [14]. Likewise, LVO was identified in 19% of the overall cohort and in 40% of patients with confirmed ischemic stroke, consistent with proportions reported in previous prospective prehospital cohorts [15,16]. These similarities provide context for the observed event rates, although they do not imply that our cohort is representative of all patients evaluated by EMS. Nevertheless, 42% of patients were ultimately diagnosed with stroke mimics, confirming the substantial diagnostic uncertainty that characterizes prehospital stroke assessment. Similar proportions have been reported in previous real-world cohorts of patients evaluated through prehospital stroke pathways [14,15]. Importantly, the consequences of this diagnostic uncertainty may extend beyond the initial assessment. In a previous cohort of EMS-transported patients to a CSC, approximately 40% of those ultimately diagnosed with a stroke mimic required hospital admission [14]. Although our study did not evaluate the appropriateness of destination decisions or resource utilization, these findings highlight the potential organizational implications of stroke mimics for CSC, where activation of stroke pathways requires rapid mobilization of specialized personnel and imaging resources.

From a clinical perspective, CPSS categories identified groups with different observed risk profiles. Nearly one-third of the cohort presented with a single abnormal CPSS component and were more frequently diagnosed with stroke mimics than stroke, with 6% harboring an LVO. In contrast, CPSS = 3 was associated with the highest observed proportions of stroke and LVO and also included a substantial proportion of ICH. This gradient is consistent with previous evidence linking greater CPSS severity to a higher likelihood of LVO [17]. Importantly, however, these findings should not be interpreted as establishing a specific transport threshold. CPSS severity alone does not distinguish ischemic stroke from ICH, and the implications of different risk levels for destination decisions depend on the clinical characteristics of individual patients in relation to the organization of the local stroke system.

The predefined CPSS groupings provided information complementary to that conveyed by CPSS categories. In our cohort, the CPSS ≥ 2 grouping was associated with an increased observed risk of LVO and showed an AUC of 0.669 (Supplement Table S1). These findings were broadly consistent with those reported in a previous retrospective cohort of patients with confirmed ischemic stroke, in which CPSS ≥ 2 showed an AUC of 0.63, with a sensitivity of 53% and specificity of 74% [18].

Our analysis also explored alternative predefined CPSS groupings based on different combinations of CPSS components. For LVO, CPSS = 3 and the CPSS 3 or 2 (F + A) grouping showed similar AUCs, while differences among the CPSS = 2 symptom patterns were small. Among patients with CPSS = 2, the Face + Arm pattern showed the highest observed probability of any stroke, whereas the groupings incorporating arm drift (“CPSS 3 or 2 F + A” and “CPSS 3 or 2 A + S”) showed slightly higher absolute risks of LVO than the Face + Speech grouping. Overall, these findings support a gradient of increasing stroke and LVO risk according to CPSS severity more clearly than the superiority of any specific symptom pattern or predefined CPSS grouping. The small differences observed among groupings should therefore be considered exploratory and require confirmation in independent cohorts.

A possible explanation for these findings relates to both the reliability and the pathophysiological significance of motor deficits. Previous studies have demonstrated that upper limb drift is the CPSS component with the highest inter-rater agreement among prehospital providers [19]. In addition, arm drift reflects dysfunction of the corticospinal tract, which is frequently involved in anterior circulation strokes, particularly those affecting the middle cerebral artery territory and proximal arterial occlusions [20]. Together, these factors may explain why symptom patterns and predefined CPSS groupings incorporating arm drift tended to identify patients at higher risk of stroke and LVO. However, because our study was not designed to evaluate the diagnostic accuracy of individual CPSS items or inter-rater agreement between EMS providers, it is not possible to determine whether the observed differences primarily reflect improved recognition of motor deficits, their greater neurological specificity, or a combination of both.

The potential relevance of symptom patterns for prehospital risk stratification has also been explored in previous studies using the CPSS for LVO identification and destination decisions. Previous work has evaluated CPSS thresholds for LVO identification and, in some settings, for direct transport to a CSC [18,21]. These studies illustrate the potential use of CPSS information beyond initial stroke recognition, while also highlighting the limitations of relying on a single threshold to identify LVO. More broadly, comparative studies have shown that no single prehospital LVO scale consistently provides excellent discrimination across different populations and settings [10,11]. Notably, the motor component of the CPSS has also been incorporated as a decision criterion in prehospital stroke triage systems, including for the selection of patients for further assessment and potential direct transport to a CSC [19].

Our findings provide a complementary perspective by describing the gradient of observed stroke and LVO risk across CPSS categories and symptom patterns rather than proposing a specific transport threshold. The differences observed among predefined CPSS groupings were small and should not be interpreted as establishing the superiority of any particular symptom combination. The clinical value of a predefined CPSS grouping depends not only on overall discrimination but also on the sensitivity-specificity trade-off associated with the selected threshold, which should be interpreted according to its intended use and the prevalence of LVO in the target population [22]. Instead, the quantitative risk estimates provided by CPSS categories and predefined CPSS groupings may help contextualize prehospital stroke and LVO risk within existing stroke pathways. Their clinical interpretation depends on the intended use of the classification, including whether the priority is to minimize missed LVO or limit unnecessary referral, as well as on factors such as transport times, access to thrombectomy-capable centers, and the organization of local EMS and stroke systems.

Several limitations should be considered. First, the reported estimates are conditional on a selected CPSS-positive stroke-code cohort. Patients with CPSS = 0 were excluded by design; therefore, the study cannot estimate the sensitivity, specificity, or negative predictive value of CPSS as a screening instrument in the overall EMS population. Limiting the cohort to CPSS-positive patients also narrowed the clinical spectrum represented and may have affected both outcome prevalence and discrimination. Second, the study specifically focused on patients assessed by BLS crews and transported to a single CSC. Patients transported to other hospitals were not included because complete hospital and imaging outcome data were not available. These characteristics limit the generalizability of the observed stroke and LVO proportions to populations with different clinical spectra or healthcare settings, while the direction of their effect on comparisons among CPSS patterns cannot be determined from the available data.

Third, 15 patients were excluded because the corresponding hospital records could not be retrieved. Because their final diagnoses were unavailable, it was not possible to determine whether their outcome distribution differed from that of the included cohort. Selection bias resulting from these exclusions therefore cannot be excluded, although these exclusions represented a small proportion of the overall cohort.

Fourth, according to the prespecified data-collection protocol, TIA and ischemic strokes were recorded within a single aggregated ischemic category. In the prehospital setting, TIA cannot be reliably distinguished from acute ischemic stroke at the time of assessment, as the distinction is established retrospectively during the hospital diagnostic evaluation.

Finally, the study was conducted within a specific regional EMS and stroke-care system and at a single CSC, and the findings have not undergone external validation in other healthcare systems. The study did not evaluate transport decisions, access to reperfusion treatment, treatment times, functional outcomes, or the consequences of false-positive and false-negative classifications. The findings therefore cannot establish the clinical utility of predefined CPSS groupings for destination triage or patient management.

Concluding, among CPSS-positive stroke-code patients assessed by BLS crews and transported to a single CSC, higher CPSS scores were associated with progressively higher observed proportions of stroke and LVO. Differences between CPSS = 2 symptom patterns were small, and the findings support a CPSS severity gradient more clearly than the superiority of any specific symptom combination. Discrimination across the evaluated CPSS categories and predefined CPSS groupings was limited to modest. These conditional estimates require external validation and should not be used alone to define transport pathways; their clinical application requires consideration of threshold-specific diagnostic performance, the consequences of misclassification, and patient-management and clinical outcomes.

## Figures and Tables

**Table 1:** Description of population and CPSS categories.

Characteristic	Overall	No Stroke	ICH	Ischemic	
				Non-LVO	LVO
N, (%)	876 (100%)	369 (42%)	96 (11%)	247 (28%)	164 (19%)
Age, years, mean ± SD	73 ± 15	70 ± 17	72 ± 15	76 ± 14	76 ± 12
Male sex	462 (53%)	183 (50%)	66 (69%)	135 (55%)	78 (48%)
CPSS score					
CPSS 1	269 (31%)	159 (43%)	15 (16%)	78 (32%)	17 (10%)
CPSS 2	319 (36%)	148 (40%)	28 (29%)	99 (40%)	44 (27%)
CPSS 3	288 (33%)	62 (17%)	53 (55%)	70 (28%)	103 (63%)
CPSS symptom patterns					
CPSS 2 (F + A)	80 (9.1%)	30 (8.1%)	12 (13%)	24 (9.7%)	14 (8.5%)
CPSS 2 (S + F)	130 (15%)	64 (17%)	3 (3%)	50 (20%)	13 (7.9%)
CPSS 2 (A + S)	109 (12%)	54 (15%)	13 (14%)	25 (10%)	17 (10%)
CPSS groupings					
CPSS ≥ 2	607 (69%)	210 (57%)	81 (84%)	169 (68%)	147 (90%)
CPSS 3 or 2 (F + A)	368 (42%)	92 (25%)	65 (68%)	94 (38%)	117 (71%)
CPSS 3 or 2 (S + F)	418 (48%)	126 (34%)	56 (58%)	120 (49%)	116 (71%)
CPSS 3 or 2 (A + S)	397 (45%)	116 (31%)	66 (69%)	95 (38%)	120 (73%)

Data are reported as absolute number (%) or mean ± SD. Percentages are expressed as proportions of the total number of cases in the corresponding column category. The ischemic stroke category includes a small proportion of patients with TIA, as specified in the study protocol. ICH = intracerebral hemorrhage; LVO = Large Vessel Occlusion; CPSS = Cincinnati Prehospital Stroke Scale. F + A = Face droop + Arm drift; S + F = Speech disturbance + Face droop; A + S = Arm drift + Speech disturbance.

**Table 2:** Absolute risk and risk ratio by CPSS categories for stroke.

CPSS Categories	Events, n/N	Absolute Risk	Risk Ratio
CPSS score			
CPSS 1	110/269	41% (35–47)	1.00
CPSS 2	171/319	54% (48–59)	1.31 (1.10–1.56)
CPSS 3	226/288	79% (73–83)	1.92 (1.64–2.24)
CPSS symptom patterns			
CPSS 2 (F + A)	50/80	62% (51–73)	1.53 (1.22–1.91)
CPSS 2 (S + F)	66/130	51% (42–60)	1.24 (0.99–1.55)
CPSS 2 (A + S)	55/109	50% (41–60)	1.23 (0.98–1.56)
CPSS groupings			
CPSS ≥ 2	397/607	65% (61–69)	1.60 (1.37–1.87)
CPSS 3 or 2 (F + A)	276/368	75% (70–79)	1.83 (1.57–2.14)
CPSS 3 or 2 (S + F)	292/418	70% (65–74)	1.71 (1.46–2.00)
CPSS 3 or 2 (A + S)	281/397	71% (66–75)	1.73 (1.48–2.03)

Stroke = ischemic stroke + intracerebral hemorrhage. CPSS = Cincinnati Prehospital Stroke Scale. The ischemic stroke category includes a small proportion of patients with TIA, as specified in the study protocol. F + A = Face droop + Arm drift; S + F = Speech disturbance + Face droop; A + S = Arm drift + Speech disturbance. CPSS groupings partially overlap and were not directly compared. Risk ratios for these groupings express the observed outcome risk relative to the common CPSS = 1 baseline and do not represent comparisons between all grouping-positive and grouping-negative participants.

**Table 3:** Absolute risk and risk ratio by CPSS categories for ischemic stroke.

CPSS Categories	Events, n/N	Absolute Risk	Risk Ratio
CPSS score			
CPSS 1	95/269	35% (30–41)	1.00
CPSS 2	143/319	45% (39–50)	1.27 (1.04–1.55)
CPSS 3	173/288	60% (54–66)	1.70 (1.41–2.05)
CPSS symptom patterns			
CPSS 2 (F + A)	38/80	47% (36–59)	1.34 (1.02–1.78)
CPSS 2 (S + F)	63/130	48% (40–57)	1.37 (1.08–1.74)
CPSS 2 (A + S)	42/109	38% (29–48)	1.09 (0.82–1.45)
CPSS groupings			
CPSS ≥ 2	316/607	52% (48–56)	1.47 (1.23–1.76)
CPSS 3 or 2 (F + A)	211/368	57% (52–62)	1.62 (1.35–1.95)
CPSS 3 or 2 (S + F)	236/418	56% (52–61)	1.60 (1.33–1.92)
CPSS 3 or 2 (A + S)	215/397	54% (49–59)	1.53 (1.27–1.85)

CPSS = Cincinnati Prehospital Stroke Scale. The ischemic stroke category includes a small proportion of patients with TIA, as specified in the study protocol. F + A = face droop + arm drift; S + F = speech disturbance + face droop; A + S = arm drift + speech disturbance. CPSS groupings partially overlap and were not directly compared. Risk ratios for these groupings express the observed outcome risk relative to the common CPSS = 1 baseline and do not represent comparisons between all grouping-positive and grouping-negative participants.

**Table 4:** Absolute and risk ratio by CPSS categories for large vessel occlusion.

CPSS Categories	Events, n/N	Absolute Risk	Risk Ratio
CPSS score			
CPSS 1	17/269	6% (4–10)	1.00
CPSS 2	44/319	14% (10–18)	2.18 (1.28–3.73)
CPSS 3	103/288	36% (30–42)	5.66 (3.48–9.20)
CPSS symptom patterns			
CPSS 2 (F + A)	14/80	17% (10–28)	2.77 (1.43–5.37)
CPSS 2 (S + F)	13/130	10% (5–16)	1.58 (0.79–3.16)
CPSS 2 (A + S)	17/109	16% (9–24)	2.47 (1.31–4.65)
CPSS groupings			
CPSS ≥ 2	147/607	24% (21–28)	3.83 (2.37–6.20)
CPSS 3 or 2 (F + A)	117/368	32% (27–37)	5.03 (3.10–8.16)
CPSS 3 or 2 (S + F)	116/418	28% (23–32)	4.39 (2.70–7.13)
CPSS 3 or 2 (A + S)	120/397	30% (26–35)	4.78 (2.95–7.76)

CPSS = Cincinnati Prehospital Stroke Scale; F + A = Face droop + Arm drift; S + F = Speech disturbance + Face droop; A + S = Arm drift + Speech disturbance. CPSS groupings partially overlap and were not directly compared. Risk ratios for these groupings express the observed outcome risk relative to the common CPSS = 1 baseline and do not represent comparisons between all grouping-positive and grouping-negative participants.

**Table 5:** Diagnostic accuracy of CPSS groupings across outcomes.

	Group	N	Prev	Se	Sp	PPV	NPV	J
*Stroke*	CPSS ≥ 2	876	0.579	0.783	0.431	0.654	0.591	0.214
	CPSS 3 or 2 (F + A)	876	0.579	0.544	0.751	0.750	0.545	0.295
	CPSS 3 or 2 (S + F)	876	0.579	0.576	0.659	0.699	0.531	0.234
	CPSS 3 or 2 (A + S)	876	0.579	0.554	0.686	0.708	0.528	0.240
	CPSS = 3	876	0.579	0.446	0.832	0.785	0.522	0.278
*Ischemic stroke*	CPSS ≥ 2	876	0.469	0.769	0.374	0.521	0.647	0.143
	CPSS 3 or 2 (F + A)	876	0.469	0.513	0.662	0.573	0.606	0.176
	CPSS 3 or 2 (S + F)	876	0.469	0.574	0.609	0.565	0.618	0.183
	CPSS 3 or 2 (A + S)	876	0.469	0.523	0.609	0.542	0.591	0.132
	CPSS = 3	876	0.469	0.421	0.753	0.601	0.595	0.174
*ICH*	CPSS ≥ 2	876	0.110	0.844	0.326	0.133	0.944	0.169
	CPSS 3 or 2 (F + A)	876	0.110	0.677	0.612	0.177	0.939	0.289
	CPSS 3 or 2 (S + F)	876	0.110	0.583	0.536	0.134	0.913	0.119
	CPSS 3 or 2 (A + S)	876	0.110	0.688	0.576	0.166	0.937	0.263
	CPSS = 3	876	0.110	0.552	0.699	0.184	0.927	0.251
*LVO*	CPSS ≥ 2	876	0.187	0.896	0.354	0.242	0.937	0.250
	CPSS 3 or 2 (F + A)	876	0.187	0.713	0.647	0.318	0.907	0.361
	CPSS 3 or 2 (S + F)	876	0.187	0.707	0.576	0.278	0.895	0.283
	CPSS 3 or 2 (A + S)	876	0.187	0.732	0.611	0.302	0.908	0.343
	CPSS = 3	876	0.187	0.628	0.740	0.358	0.896	0.368

CPSS = Cincinnati Prehospital Stroke Scale; Prev = prevalence; Se = sensitivity; Sp = specificity; PPV = positive predictive value; NPV = negative predictive value; J = Youden index; the ischemic stroke category includes a small proportion of patients with TIA, as specified in the study protocol. ICH = hemorrhagic stroke; LVO = large vessel occlusion; F + A = Face droop + Arm drift; S + F = Speech disturbance + Face droop; A + S = Arm drift + Speech disturbance. Stroke includes ischemic and hemorrhagic stroke. All outcomes, including LVO, use the full included cohort (N = 876) as denominator, the included cohort was restricted to CPSS-positive patients.

## Data Availability

Original data are available upon reasonable request to the corresponding author.

## Supplementary Materials

The following supporting information can be downloaded at: https://www.mdpi.com/article/10.3390/jcm15176781/s1, Supplementary Materials S1: Inclusion and exclusion criteria; 2. Table S1: CPSS categories and stroke outcomes reporting odds ratios and AUC; 3. Figure S1: decision curve analysis; 4. Figure S2: heatmap.
