# Supplementary material for: Stroke and Large Vessel Occlusion Risk According to Cincinnati Prehospital Stroke Scale Symptom Patterns in a CPSS-Positive EMS Cohort: A Prospective Cohort Study
Source: J Clin Med. 2026 Sep 1;15(17):6781. doi: 10.3390/jcm15176781 (PMC13566748; doi:10.3390/jcm15176781)
Supplement: Supplementary file 1 [file jcm-15-06781-s001.zip › Figure S1.pdf]

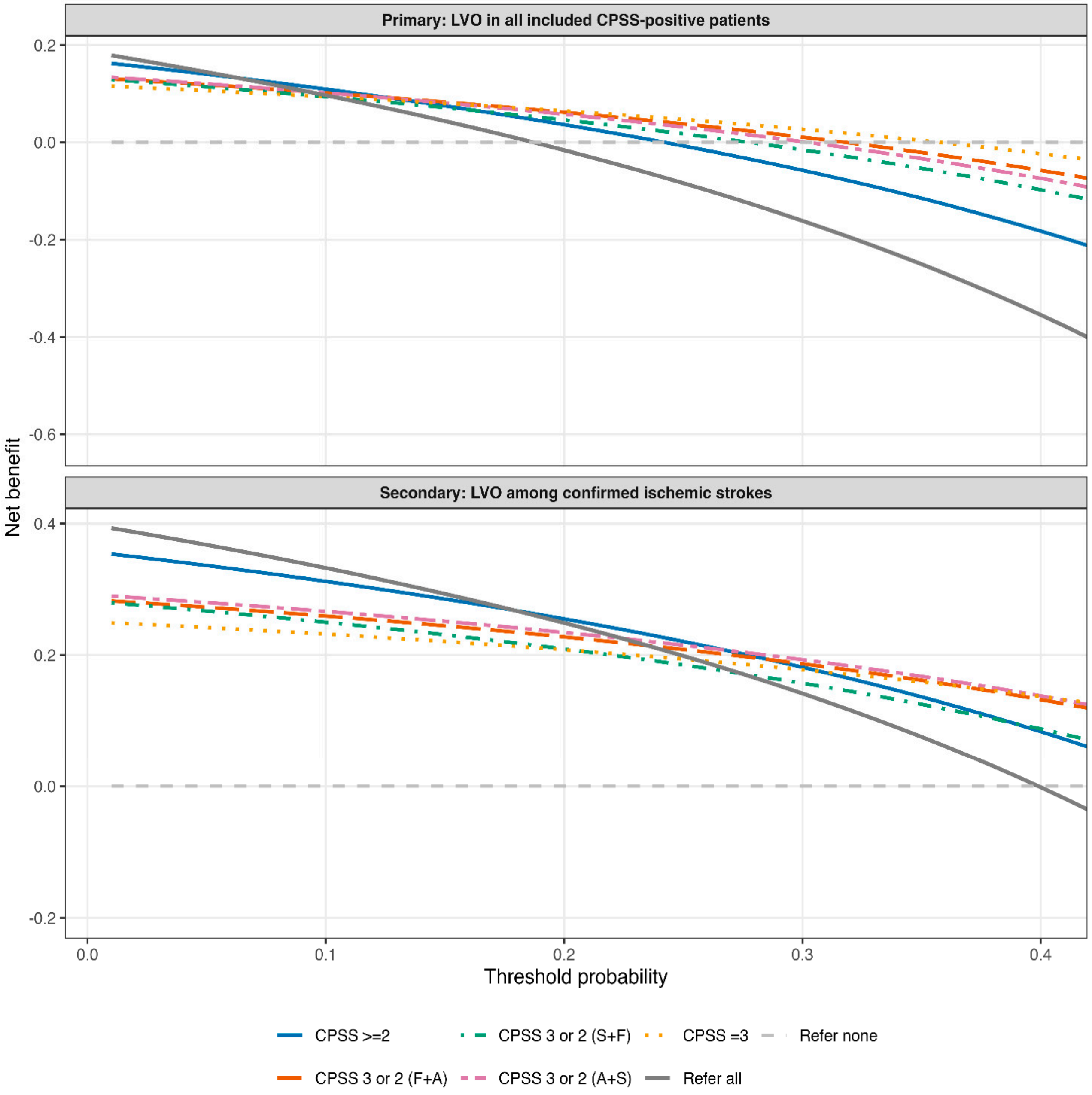

Fixed-rule DCA: net benefit = TP/N - FP/N x Pt/(1-Pt). The primary cohort contains only patients with CPSS >=1.

Figure S1. Decision curve analysis (DCA) of different predefined Cincinnati Prehospital Stroke Scale (CPSS) categories for predicting large vessel occlusion (LVO). The upper panel shows the analysis performed in the overall study population, whereas the lower panel includes only patients with confirmed ischemic stroke. The x-axis represents the threshold probability (Pt), reflecting the trade-off between true-positive and false-positive classifications, whereas the y-axis shows the net benefit (NB). A CPSS model was considered clinically useful when its net benefit exceeded that of the default "treat all" and "treat none" strategies across a given range of threshold probabilities. The ischemic stroke category includes a small proportion of patients with TIA, as specified in the study protocol. Abbreviations: CPSS, Cincinnati Prehospital Stroke Scale; LVO,

large vessel occlusion; F+A, face droop plus arm drift; S+F, speech disturbance plus face droop; A+S, arm drift plus speech disturbance.
