# Supplementary material for: Stroke and Large Vessel Occlusion Risk According to Cincinnati Prehospital Stroke Scale Symptom Patterns in a CPSS-Positive EMS Cohort: A Prospective Cohort Study
Source: J Clin Med. 2026 Sep 1;15(17):6781. doi: 10.3390/jcm15176781 (PMC13566748; doi:10.3390/jcm15176781)
Supplement: Supplementary file 1 [file jcm-15-06781-s001.zip › Figure S2.pdf]

Pairwise Delta AUC heatmaps - Stroke

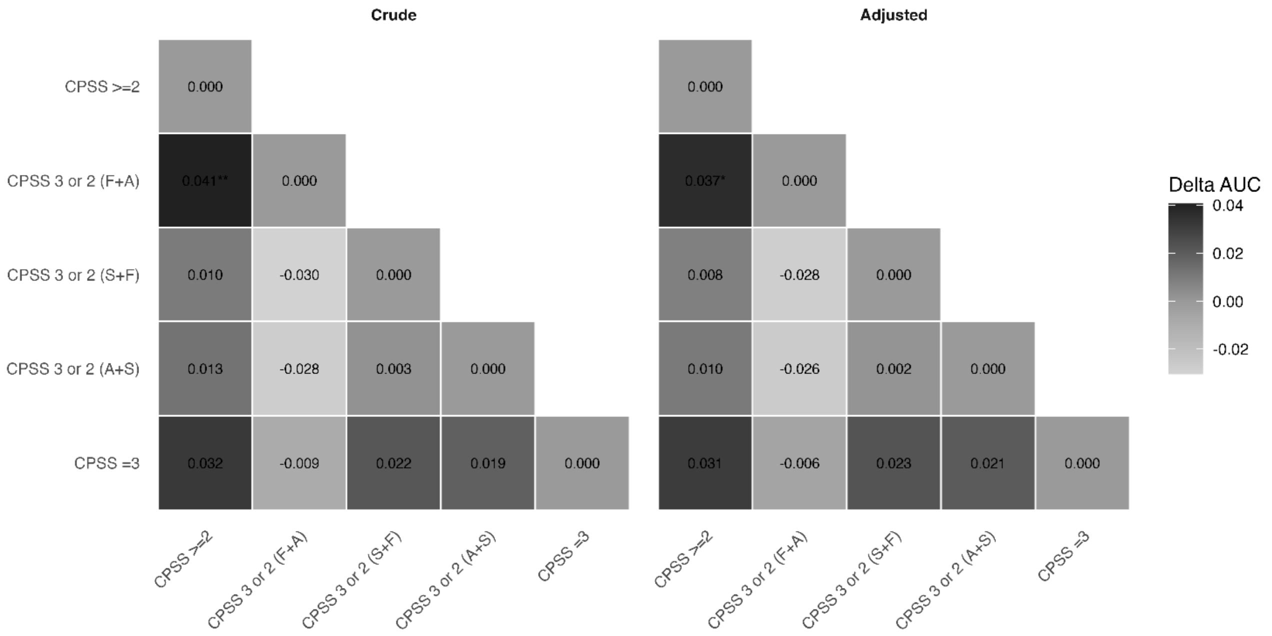

Positive values indicate higher AUC for the row model. * p<0.05; ** p<0.01; *** p<0.001.

Pairwise Delta AUC heatmaps - Ischemic stroke

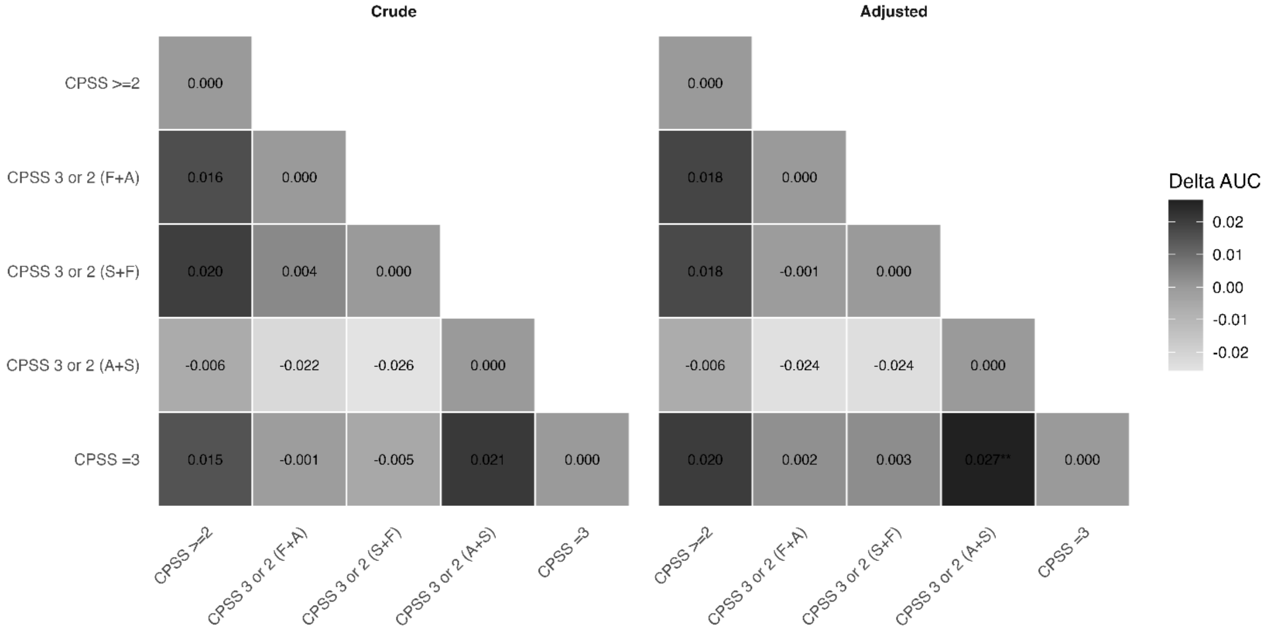

Positive values indicate higher AUC for the row model. * p<0.05; ** p<0.01; *** p<0.001.

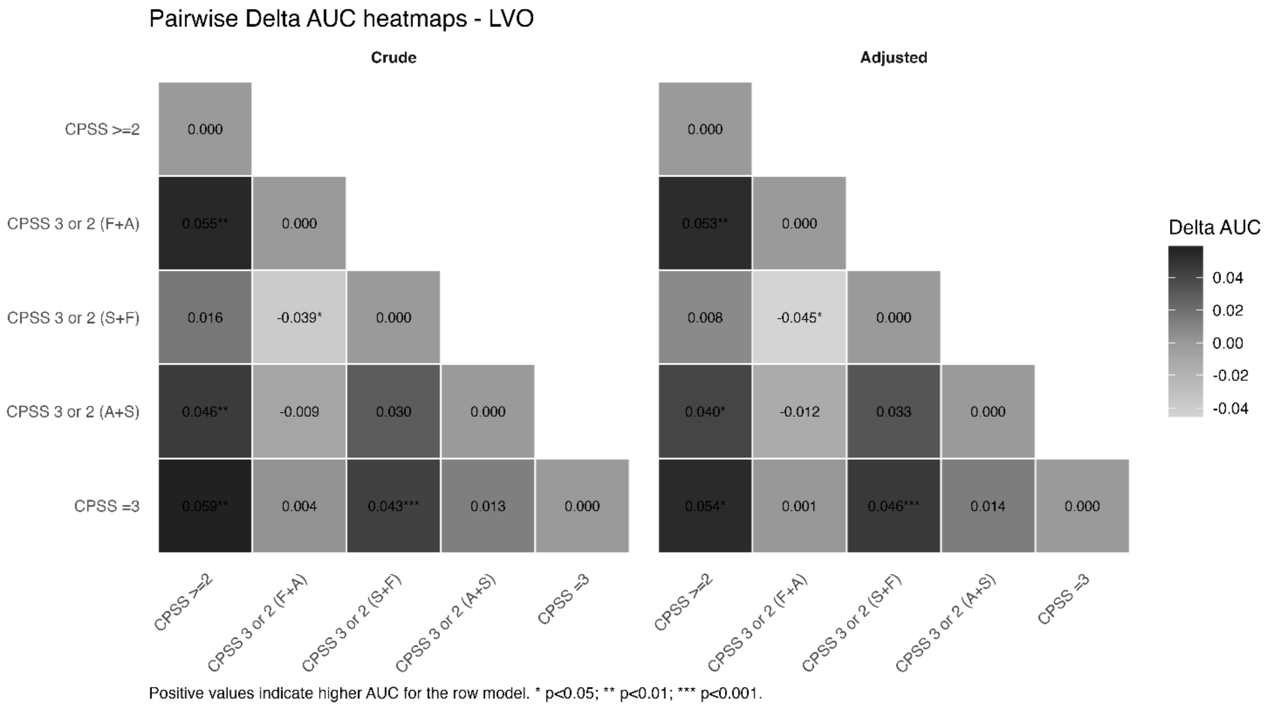

Figure S2. The heatmaps are presented in the following order: stroke (ischemic stroke and intracerebral hemorrhage), ischemic stroke and large vessel occlusion (LVO). They show the area under the receiver operating characteristic curve (AUC) for the different predefined Cincinnati Prehospital Stroke Scale (CPSS) groupings used to predict stroke, ischemic stroke and LVO. Color intensity indicates categories discrimination and supports comparison of diagnostic performance across predefined CPSS groupings.The ischemic stroke category includes a small proportion of patients with TIA, as specified in the study protocol. F+A = Face droop + Arm drift; S+F = Speech disturbance + Face droop; A+S = Arm drift + Speech disturbance.
