# Supplementary material for: Stroke and Large Vessel Occlusion Risk According to Cincinnati Prehospital Stroke Scale Symptom Patterns in a CPSS-Positive EMS Cohort: A Prospective Cohort Study
Source: J Clin Med. 2026 Sep 1;15(17):6781. doi: 10.3390/jcm15176781 (PMC13566748; doi:10.3390/jcm15176781)
Supplement: Supplementary file 1 [file jcm-15-06781-s001.zip › Supplementary material S1.pdf]

Supplementary material S1

Inclusion criteria:

- Age ≥ 18 years
- Management by a Basic Life Support (BLS) crew
- Alert or Verbal on neurological assessment with AVPU scale
- Completion and documentation of the CPSS[1] in the EMS[2] electronic record
- Assignment of a stroke code by the SOREU[3] Metropolitana during the prehospital assessment

  [A Stroke code is a patient with at least 1 point of CPSS, known or presumed onset of symptoms within 24 hours and a modified Rankin Scale ≤3]
- Transport and admission to the Grande Ospedale Metropolitano Niguarda
- Availability of a confirmed final hospital diagnosis (ischemic stroke, hemorrhagic stroke, transient ischemic attack, or alternative diagnosis)

Exclusion criteria:

- Age < 18 years;
- Cases managed by Advanced Life Support units
- Incomplete or missing prehospital CPSS documentation
- No stroke code assigned
- Missing or indeterminate final hospital diagnosis

[1] CPSS = Cincinnati Prehospital Stroke Scale; [2]EMS = Emergency Medical Service; [3]SOREU = Sala Operativa Regionale Emergenza Urgenza of the Metropolitan Area of Milan
