# Supplementary material for: Stroke and Large Vessel Occlusion Risk According to Cincinnati Prehospital Stroke Scale Symptom Patterns in a CPSS-Positive EMS Cohort: A Prospective Cohort Study
Source: J Clin Med. 2026 Sep 1;15(17):6781. doi: 10.3390/jcm15176781 (PMC13566748; doi:10.3390/jcm15176781)
Supplement: Supplementary file 1 [file jcm-15-06781-s001.zip › Table S1.pdf]

Outcome	Categories	Specification	OR (95% CI)	AUC
**Stroke**	CPSS >=2	Crude	2.73 (2.03-3.67)	0.607
**Stroke**	CPSS 3 or 2 (F+A)	Crude	3.60 (2.68-4.82)	0.648
**Stroke**	CPSS 3 or 2 (S+F)	Crude	2.62 (1.98-3.46)	0.617
**Stroke**	CPSS 3 or 2 (A+S)	Crude	2.71 (2.05-3.59)	0.620
**Stroke**	CPSS =3	Crude	3.98 (2.88-5.51)	0.639
**Stroke**	CPSS >=2	Adjusted	2.75 (2.03-3.72)	0.663
**Stroke**	CPSS 3 or 2 (F+A)	Adjusted	3.78 (2.79-5.11)	0.699
**Stroke**	CPSS 3 or 2 (S+F)	Adjusted	2.64 (1.99-3.51)	0.671
**Stroke**	CPSS 3 or 2 (A+S)	Adjusted	2.75 (2.06-3.66)	0.673
**Stroke**	CPSS =3	Adjusted	4.12 (2.96-5.75)	0.694
**Ischemic stroke**	CPSS >=2	Crude	1.99 (1.48-2.68)	0.572
**Ischemic stroke**	CPSS 3 or 2 (F+A)	Crude	2.07 (1.58-2.72)	0.588
**Ischemic stroke**	CPSS 3 or 2 (S+F)	Crude	2.10 (1.60-2.75)	0.591
**Ischemic stroke**	CPSS 3 or 2 (A+S)	Crude	1.71 (1.30-2.23)	0.566
**Ischemic stroke**	CPSS =3	Crude	2.21 (1.66-2.95)	0.587
**Ischemic stroke**	CPSS >=2	Adjusted	1.99 (1.47-2.69)	0.629
**Ischemic stroke**	CPSS 3 or 2 (F+A)	Adjusted	2.18 (1.64-2.88)	0.648
**Ischemic stroke**	CPSS 3 or 2 (S+F)	Adjusted	2.11 (1.60-2.78)	0.647
**Ischemic stroke**	CPSS 3 or 2 (A+S)	Adjusted	1.72 (1.31-2.27)	0.623
**Ischemic stroke**	CPSS =3	Adjusted	2.29 (1.71-3.08)	0.650
**LVO**	CPSS >=2	Crude	4.74 (2.80-8.01)	0.625
**LVO**	CPSS 3 or 2 (F+A)	Crude	4.57 (3.15-6.63)	0.680
**LVO**	CPSS 3 or 2 (S+F)	Crude	3.28 (2.27-4.74)	0.642
**LVO**	CPSS 3 or 2 (A+S)	Crude	4.28 (2.94-6.24)	0.671
**LVO**	CPSS =3	Crude	4.81 (3.36-6.88)	0.684
**LVO**	CPSS >=2	Adjusted	4.75 (2.80-8.04)	0.669
**LVO**	CPSS 3 or 2 (F+A)	Adjusted	4.92 (3.37-7.19)	0.722
**LVO**	CPSS 3 or 2 (S+F)	Adjusted	3.29 (2.27-4.76)	0.677
**LVO**	CPSS 3 or 2 (A+S)	Adjusted	4.40 (3.01-6.43)	0.709
**LVO**	CPSS =3	Adjusted	5.10 (3.54-7.35)	0.723

Table S1. Logistic regression analysis of different CPSS categories for predicting stroke (ischemic stroke + intracerebral hemorrhage), ischemic stroke, and large vessel occlusion (LVO). The ischemic stroke category includes a small proportion of patients with TIA, as specified in the study protocol. All associations were

statistically significant ($p<0.001$). CPSS = Cincinnati Prehospital Stroke Scale. F+A = Face droop + Arm drift; S+F = Speech disturbance + Face droop; A+S = Arm drift + Speech disturbance. Crude estimates are unadjusted; adjusted estimates are adjusted for age and sex. Age was modeled as a continuous variable. Regression analyses were exploratory association analyses and were not intended to develop clinical prediction models. AUCs are reported with 95% CIs.
